# Opioid use and naloxone-identified suspected opioid-related adverse events in a tertiary pediatric hospital: a retrospective study

**DOI:** 10.1016/j.jped.2026.101628

**Published:** 2026-10-30

**Authors:** Luíza Siqueira Lima, Meire Ellen Pereira, Júlia Vicentin de Souza, Diancarlos Pereira de Andrade, Cristian Rodolfo Voigt, Julliana Bianco Giuriatti Bassani, Carolina Prando, Cristiano Matos, João José Joaquim, Cláudia Sirlene Oliveira

**Affiliations:** a Instituto de Pesquisa Pelé Pequeno Príncipe, Curitiba, PR, Brazil; b Faculdades Pequeno Príncipe, Curitiba, PR, Brazil; c Hospital Pequeno Príncipe, Curitiba, PR, Brazil; d Centro de Investigação, Inovação e Desenvolvimento em Enfermagem de Lisboa (CIDNUR), Escola Superior de Enfermagem, Universidade de Lisboa, Lisboa, Portugal; e Instituto de Saúde Ambiental (ISAMB), Faculdade de Medicina, Universidade de Lisboa, Lisboa, Portugal; f Instituto Politécnico de Coimbra, ESTESC-Coimbra Health School, Farmácia, Coimbra, Portugal

**Keywords:** Analgesics, opioid, Child, Drug-related side effects and adverse reactions, Naloxone, Retrospective studies

## Abstract

**Objective:**

Opioids are essential for analgesia and sedation in pediatric inpatient care, particularly in perioperative and intensive care settings. However, their use requires careful monitoring because children, especially infants and critically ill patients, may be vulnerable to respiratory, cardiovascular, and neurological adverse effects.

**Methods:**

This retrospective observational study evaluated opioid administration records and naloxone-identified suspected opioid-related adverse-event episodes at a tertiary pediatric hospital in Brazil from January 2019 to September 2024. Opioid administration records were identified from hospital pharmacy dispensing data. Medical records of patients with naloxone administration records were reviewed to classify naloxone indication, opioid involvement, clinical manifestations, hospital setting, underlying conditions, and short-term outcomes.

**Results:**

During the study period, 154,867 opioid administration records were identified. A total of 284 naloxone administration records were screened. After medical record review, 171 naloxone administrations did not meet criteria for suspected opioid-related adverse-event episodes and were excluded from the main adverse-event analysis. The final naloxone-identified sample included 112 episodes. Events occurred predominantly in intensive care settings, especially the cardiac intensive care unit. The most frequently documented manifestations were bradycardia, oxygen desaturation, apnea, chest wall rigidity, drowsiness/somnolence, and worsened respiratory pattern. Most episodes improved after naloxone administration.

**Conclusion:**

Naloxone-identified suspected opioid-related adverse-event episodes were uncommon relative to the total volume of opioid administration records and were concentrated in clinically complex pediatric inpatients. These findings support risk-stratified monitoring, standardized documentation of naloxone indications, and continued opioid stewardship in tertiary pediatric care.

## Introduction

Opioids are widely used in pediatric inpatient care for analgesia and sedation, particularly in perioperative and intensive care settings. Although these medications remain essential for pain control and procedural management, their use requires careful monitoring in pediatric patients [1]. In hospital settings, naloxone is used to reverse opioid-induced respiratory or central nervous system depression [2]. Naloxone may also be used outside opioid-related adverse-event contexts, reinforcing the need for medical record review to classify its clinical indication [3]. Nevertheless, its antagonistic action may also compromise analgesia, thereby increasing the risk of pain [4]. Therefore, naloxone use may serve as a pragmatic clinical trigger for identifying suspected opioid-related events [5,6]. Characterizing the frequency, patterns, and clinical consequences of naloxone-related episodes is essential for generating evidence-based strategies aimed at improving patient safety and optimizing opioid stewardship [7].

The narrow therapeutic index of many opioid agents contributes to the risk of dosing-related harm, particularly in pediatric hospital care, where dosing is often weight-based, and patients may have age-related pharmacokinetic and pharmacodynamic vulnerabilities [8], while administration-route errors remain relatively frequent [9]. Accidental pediatric opioid exposures have been described in emergency settings, but hospital-based opioid safety data in children remain comparatively limited [10]. In Brazil, pediatric data on opioid safety remain limited, particularly in hospital settings where opioids are used for analgesia, sedation, perioperative care, and intensive care management [11]. This trend is relevant to hospital care, where opioids are frequently used for analgesia, sedation, perioperative management, intensive care, and palliative care [12]. This lack of information highlights the need for hospital-based studies describing opioid use, naloxone administration, clinical manifestations, and short-term outcomes in pediatric patients. Further research is needed to characterize pediatric opioid use and safety in real-world hospital settings, particularly among clinically complex children who may be more vulnerable to respiratory, cardiovascular, and neurological complications [13].

Pediatric hospital data from Brazil remain limited, particularly on naloxone-identified suspected opioid-related adverse-event episodes. In hospital settings, naloxone may serve as a pragmatic trigger for medical record review, although it does not capture events managed without antagonist administration and may also be used in non-adverse-event contexts. Therefore, this retrospective single-center study described opioid administration patterns and characterized naloxone-identified episodes at a tertiary pediatric hospital from January 2019 to September 2024, aiming to support risk-stratified monitoring, standardized documentation, and safer opioid use in pediatric care.

## Methods

### Study design and setting

This retrospective observational study was conducted at a tertiary pediatric hospital in Curitiba, Paraná, Brazil. The hospital provides high-complexity pediatric care, including emergency care, surgical care, and neonatal, cardiac, general, and surgical intensive care units. The study was approved by the Ethics Committee of the Hospital under protocol number 6.599.719.

### Data sources and opioid administration records

Pharmacy data were obtained from the hospital pharmacy information system and included opioid stock-movement records linked to patient care between January 2019 and September 2024. Records indicating medication return to pharmacy stock were excluded; therefore, opioid administration records were operationally defined as opioid dispensing records without a subsequent return-to-stock entry. This operational definition was used as a proxy for opioid utilization and does not establish whether the dispensed medication was fully or partially administered to the patient. The pharmacy dataset included alfentanil, buprenorphine, fentanyl, methadone, morphine, naloxone, and remifentanil formulations. During medical record review of naloxone use, additional opioids not represented in the pharmacy extract, such as codeine and tramadol, were identified and included in case-level analyses.

### Eligibility criteria and naloxone-triggered medical record review

Patients were eligible for the naloxone-triggered medical record review if they had at least one naloxone administration record during the study period. All naloxone administration records were screened, and the corresponding medical records were reviewed to determine the clinical indication for naloxone use, documented signs and symptoms, opioid exposure, hospital unit, underlying condition, and outcome after naloxone administration. Although the analysis was conducted in a pediatric center, selected adolescents and young adults with chronic or complex pediatric-onset conditions may remain under follow-up in pediatric hospital services. These patients were retained in the analysis when naloxone use occurred within pediatric care units, and age distribution was reported transparently.

### Classification of naloxone use and suspected opioid-related adverse-event episodes

Suspected opioid-related adverse-event episodes were identified using naloxone administration records as a pragmatic screening trigger. All naloxone records were linked to medical records and reviewed independently by three trained reviewers using a standardized extraction form. Extracted variables included naloxone indication, opioid exposure, clinical manifestations, hospital unit, underlying condition, and short-term outcome. Episodes were classified as naloxone-identified suspected opioid-related adverse events, off-label/non-opioid naloxone use, non-confirmed opioid-related naloxone use, or not documented/not classifiable. Clinical manifestations were transcribed as recorded, without post hoc reclassification. Discrepancies were resolved by discussion and adjudication by the principal investigator. Opioid involvement was analyzed at both the episode and opioid-mention levels when multiple opioids were documented.

### Clinical manifestations and outcomes

Clinical manifestations were extracted as documented by the attending medical team in the medical record. When multiple signs or symptoms were documented in the same episode, each manifestation was recorded separately. Percentages for clinical manifestations were calculated using the number of naloxone-identified suspected opioid-related adverse-event episodes as the denominator, and categories were considered non-mutually exclusive. Short-term outcomes after naloxone administration were classified according to documented clinical response, including complete symptom resolution, partial improvement, no clinical improvement, withdrawal syndrome, death attributed to underlying disease, or not reported.

### Statistical analyses

Statistical analyses were performed using R software, version 4.3.3. Continuous variables were summarized as mean or median with standard deviation or interquartile range, as appropriate, and categorical variables as absolute and relative frequencies. Percentages were calculated using explicit denominators according to the unit of analysis: opioid administration records, naloxone records, or naloxone-identified suspected opioid-related adverse-event episodes. Clinical manifestation categories were not mutually exclusive.

## Results

### Study flow and classification of naloxone use

A total of 154,867 opioid administration records were identified between January 2019 and September 2024, after exclusion of pharmacy records with documented return to stock. During the same period, 284 naloxone administration records were screened. After medical record review, 171 naloxone administrations did not meet criteria for naloxone-identified suspected opioid-related adverse-event episodes and were excluded from the main adverse-event analysis. The final naloxone-identified sample included 112 episodes among 110 patients. Of these, 109 were classified as suspected opioid-related adverse-event episodes and three as off-label or non-confirmed opioid-related naloxone use (Figure 1).

**Figure 1:**
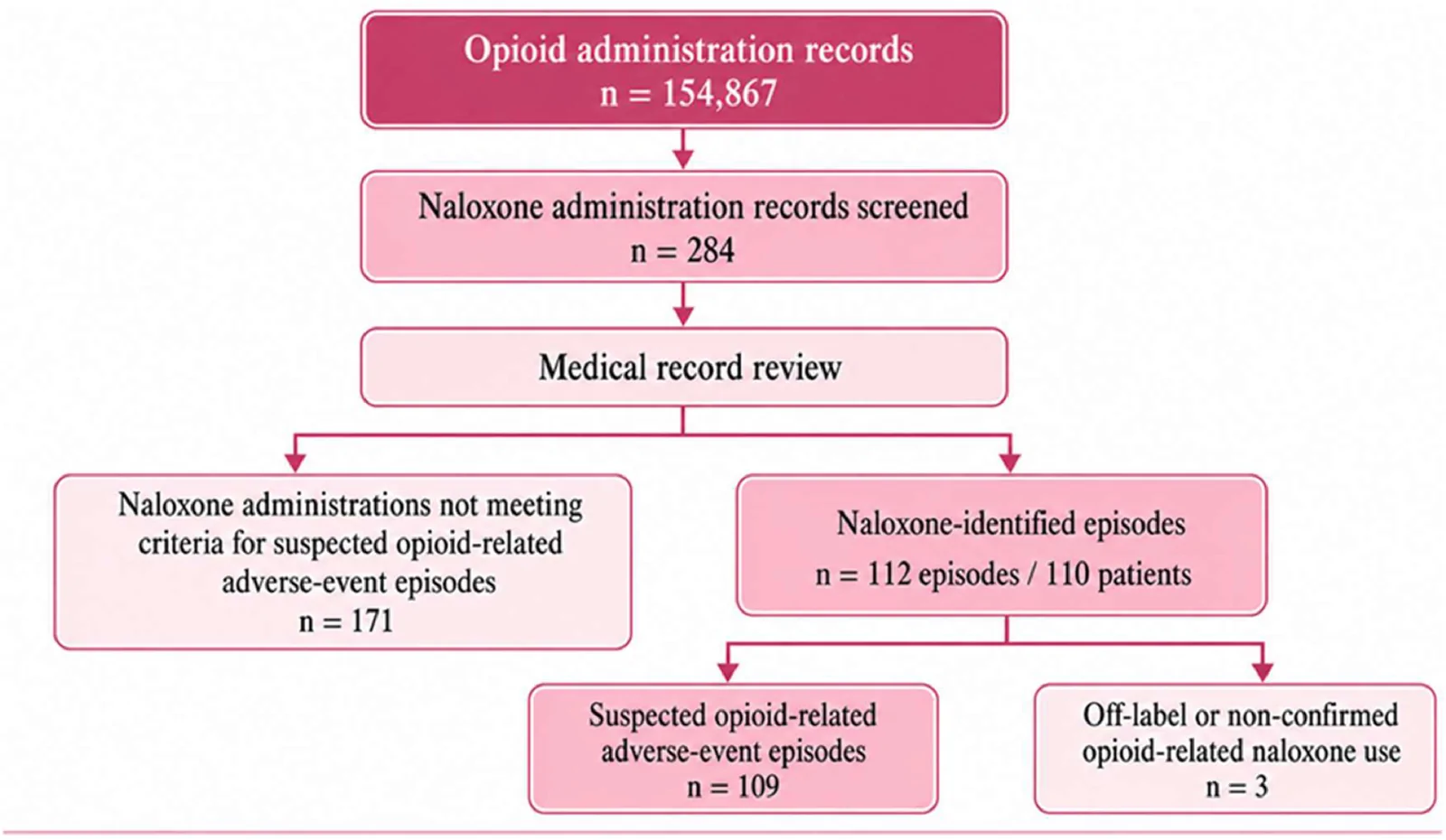
Flowchart of opioid administration records, naloxone screening, and classification of naloxone-identified episodes.

### Opioid administration records

A total of 154,867 opioid administration records were recorded between January 2019 and September 2024. The highest administration record volume was observed in 2019 (30,721; 20%), followed by 2023 (29,521; 19%), 2022 (26,166; 17%), 2021 (23,712; 15%), 2024 (22,965; 15%), and 2020 (21,782; 14%) (Fig. S1). Annual opioid administration records are shown in Figure 2.

**Figure 2:**
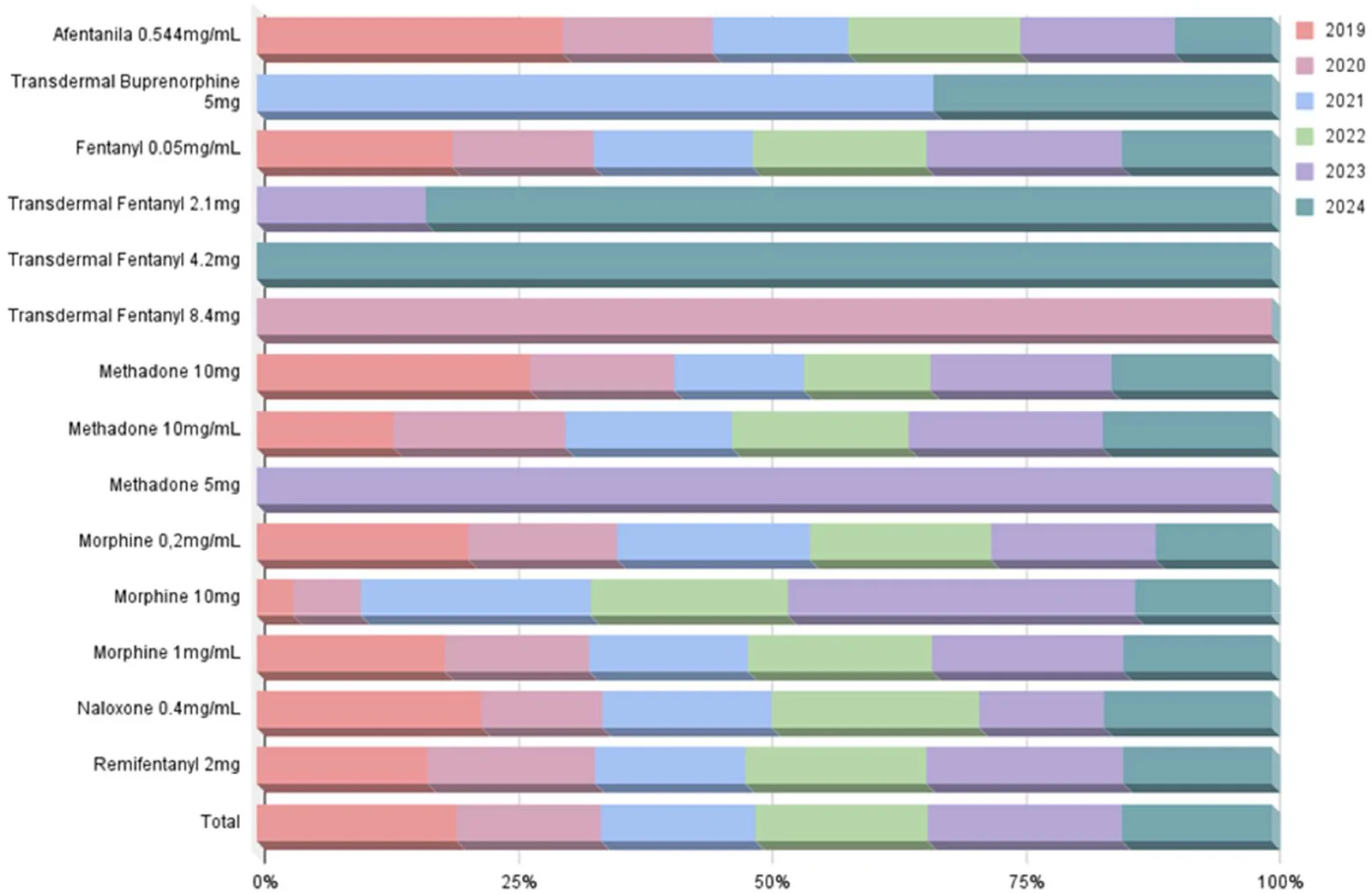
Annual distribution of opioid administration records by opioid type from January 2019 to September 2024.

Among the analyzed years, fentanyl (0.05 mg/mL) accounted for the highest proportion of administration records (43%), followed by morphine (1 mg/mL, 38%) and methadone (10 mg, 13%) (Fig. S2). In 2019, administration record volumes included 66,998 units of fentanyl 0.05 mg/mL (19% of total fentanyl administration records), 59,283 units of morphine 1 mg/mL (18% of total morphine administration records), and 19,618 units of methadone 10 mg (27% of total methadone administration records). In 2023, the distribution was similar for fentanyl (19%) and morphine (18%), whereas methadone accounted for 18% of its total administration records. Notably, administration records of morphine 10 mg increased markedly, from 38 administration records (4.2%) in 2019 to 346 (39%) in 2023 (Figure 2 and Supplementary Table 1).

Opioid administration records categorized according to the age of the patients who received them are shown in Figure S3. The highest frequency of opioid administration records was observed in the 0–2 years age group (70,164 opioid administration records), followed by 11–17 years (35,273 opioid administration records), 6–10 years (27,707 opioid administration records), 3–5 years (24,488 opioid administration records), and ≥18 years (891 opioid administration records).

### Characteristics of naloxone-identified suspected opioid-related adverse-event episodes

A total of 284 naloxone administration records were screened through medical record review. The highest proportion occurred in 2019 (23.24%), followed by 2022 (20.77%), 2021 (16.9%), 2024 (16.55%), 2023 (12.32%), and 2020 (11.97%). Of the screened records, 171 were not associated with suspected opioid-related adverse-event episodes and were documented in other clinical contexts, including sedoanalgesia reversal, postoperative recovery, intentional awakening after surgery, or planned/unplanned extubation. The final naloxone-identified sample included 112 episodes among 110 patients; 109 were classified as suspected opioid-related adverse-event episodes and three as off-label or non-confirmed opioid-related naloxone use, including exogenous intoxication or suspected but unconfirmed opioid involvement. Supplementary Table S2 presents individual-level data on opioid involvement, underlying disease, hospital unit, recorded adverse event, and outcome after naloxone use. Most episodes occurred in children aged 0–2 years (n = 70), followed by 11–17 years (n = 19), 6–10 years (n = 15), and 3–5 years (n = 8). Regarding sex, 50 episodes (45%) occurred in females and 62 (55%) in males (Fig. S4).

### Opioids involved in the related adverse events

For analysis purposes, opioid formulations were aggregated by active ingredients, pooling all concentrations and presentations for each opioid. The opioid associated with the reported adverse events is presented in Table 1. Fentanyl was the most frequently associated with suspected opioid-related adverse-event episodes, accounting for 75 of 147 opioid mentions (51.0%), followed by morphine (42/147; 28.5%) and methadone (24/147; 16%). Tramadol (2/147; 1.4%) and codeine (3/147; 2.1%) were rarely reported. In one suspected episode, the specific opioid involved could not be identified because the patient had been referred from another care center and opioid exposure could not be determined from the available records.

**Table 1:** Opioids documented in naloxone-identified suspected opioid-related adverse-event episodes during follow-up periods from January 2019 to September 2024.

	Year of administration records						
Drug⁎	2019	2020	2021	2022	2023	2024	Total
Fentanyl	19	12	14	14	8	8	75
Morphine	8	8	5	10	7	4	42
Methadone	4	4	2	8	2	4	24
Tramadol	1	0	0	1	0	0	2
Codeine	0	1	0	1	0	1	3
Not identified	0	0	0	1	0	0	1

⁎ In the way that more than one opioid could be documented in the same episode, opioid mentions exceeded the number of episodes.

When analyses were restricted to episodes with only one documented opioid, 74 episodes were identified. Fentanyl was documented in 46 episodes (62%), followed by morphine in 17 (23%) and methadone in 11 (15%). A total of 35 episodes involved exposure to more than one opioid during the adverse event episode, with the most frequent combination being fentanyl and morphine, reported in 18 cases (51%). These findings suggest possible polyexposure or concomitant administration of multiple opioids and other pharmacological agents. Fentanyl was the most frequently documented opioid among suspected adverse-event episodes; however, fentanyl was also the most frequently administered opioid during the study period. Therefore, these findings should be interpreted as the frequency of involvement rather than comparative risk. Patient-level exposure denominators for individual opioids were unavailable; therefore, opioid-specific event rates could not be calculated. Accordingly, the frequencies observed for fentanyl, morphine, and methadone should be interpreted as descriptive involvement in naloxone-identified episodes rather than as comparative incidence or risk.

### Clinical characteristics of opioid-related adverse events

After review of the medical records of patients identified through the naloxone administration records registry, opioid-related adverse events were characterized. Table 2 summarizes documented clinical manifestations by episode. Categories were not mutually exclusive because more than one manifestation could be recorded in the same episode.

**Table 2:** Clinical manifestations documented during naloxone-identified suspected opioid-related adverse-event episodes during follow-up periods from January 2019 to September 2024.

Clinical manifestation⁎	Pediatric episodes, n (%)
Bradycardia	33 (29.5)
Oxygen desaturation/decrease in SpO₂	31 (27.7)
Apnea	21 (18.8)
Chest wall rigidity	17 (15.2)
Drowsiness/somnolence	17 (15.2)
Dyspnea/worsened respiratory pattern/gasping	17 (15.2)
Hypoactivity/decreased responsiveness	13 (11.6)
Altered level of consciousness	11 (9.8)
Cyanosis	10 (8.9)
Bradypnea	9 (8.0)
Seizure	7 (6.3)
Miosis	6 (5.4)
Reduced pulmonary expansibility	6 (5.4)
Agitation	6 (5.4)
Cardiopulmonary arrest	6 (5.4)
Sedation	6 (5.4)
Vomiting	5 (4.5)
Tachycardia	4 (3.6)
Hypotension	3 (2.7)
Nausea	3 (2.7)
Fever	1 (0.9)

⁎ Categories are not mutually exclusive because more than one clinical manifestation could be documented in the same episode. Percentages were calculated using 112 naloxone-identified suspected opioid-related episodes as the denominator.

The most frequently documented clinical manifestations were bradycardia (33; 29.5%), oxygen desaturation (31; 27.7%), apnea (21; 18.8%), chest wall rigidity (17; 15.2%), drowsiness/somnolence (17; 15.2%), and dyspnea or worsened respiratory pattern/gasping (17; 15.2%).

### Hospitalization setting and baseline clinical conditions

Regarding the hospitalization setting, most patients were in the Intensive Care Units (ICUs), with a predominance of the cardiac ICU. Specifically, 48 patients were admitted to the cardiac ICU (42.8%), 21 to the surgical ICU (18.8%), 15 to the general ICU (13.4%), 7 to the neonatal ICU (6.3%), and 3 to a specialized surgical ICU (2.7%). Additionally, 10 patients (8.9%) were managed in the emergency department, whereas 8 (7.1%) were treated in general hospital wards, including neurological, pediatric, cardiac, and surgical units (Supplementary Table S2).

Among the 112 evaluated adverse event episodes, baseline hospitalization diagnoses were classified according to the International Statistical Classification of Diseases and Related Health Problems, 10th Revision (ICD-10). Supplementary Table S2 summarizes the diagnostic codes corresponding to each patient’s underlying condition at admission. Congenital heart defects were the most prevalent diagnoses, with atrial septal defect (Q21.1) identified in 17 patients (14.9%) and ventricular septal defect (Q21.0) in 14 patients (12.3%). Other congenital circulatory malformations, including congenital pulmonary valve stenosis (Q22.1) and patent ductus arteriosus (Q25.0), were each observed in 8 patients (7.0%). Secondary pulmonary hypertension (I27.2) was documented in 8 patients (7.0%), likely reflecting the hemodynamic burden associated with underlying structural cardiac anomalies. Additionally, 8 patients (7.0%) were classified as extremely low birth weight (P07.3).

### Short-term outcomes after naloxone administration

Regarding short-term outcomes after naloxone administration, complete symptom resolution was documented in 76 episodes (67.9%). Partial improvement was observed in 12 episodes (10.7%), while 8 episodes (7.1%) showed no clinical improvement. Eleven episodes (9.8%) resulted in death due to underlying conditions unrelated to naloxone administration or suspected opioid-related adverse events. Withdrawal syndrome was documented in two episodes (1.8%), and one episode (0.9%) had no reported outcome. Off-label or non-confirmed opioid-related naloxone use was identified in three episodes (2.7%), for which outcomes were not reported.

## Discussion

In this retrospective study conducted at a tertiary pediatric hospital, naloxone-identified suspected opioid-related adverse-event episodes were uncommon relative to the total number of opioid administration records. Episodes occurred predominantly in high-acuity settings, especially cardiac and surgical ICUs, and were mainly characterized by respiratory, cardiovascular, and central nervous system manifestations. Most episodes improved after naloxone administration, suggesting timely recognition and management in many cases. The observed frequency should be interpreted as the rate of naloxone-identified suspected episodes rather than the overall incidence of opioid-related adverse events, because events managed without naloxone were not captured.

Adequate pain management in infants is a clinical priority due to their immature nociceptive and central pain modulation systems. Poor pain control may negatively affect brain development and lead to long-term physiological, psychological, and neurodevelopmental consequences, highlighting the need to balance effective analgesia with careful monitoring of adverse outcomes [14]. In the present study, 112 naloxone-identified episodes were included in the case-level description, corresponding to 109 suspected opioid-related adverse-event episodes and three off-label or non-confirmed opioid-related naloxone uses.

Opioids such as fentanyl, methadone, and morphine are classified as high-alert medications (HAMs) because errors in their use carry a high risk of serious patient harm. Therefore, they require heightened vigilance, given their toxicity potential and the need for strict administration and monitoring protocols [15]. In the present study, fentanyl, morphine, and methadone accounted for the highest number of opioid administration records and were also the most frequently implicated by mention in adverse-event episodes. Similar findings were reported by Carvalho et al. [16]. a Brazilian multicenter study of 28 hospitals, which reported that tramadol, fentanyl, and morphine as the most frequently used opioids and showed that the COVID-19 pandemic altered in-hospital opioid use, particularly among patients requiring ICU care and ventilatory support. In the single-center pediatric analysis, administration records decreased in 2020 and then increased again in 2021, likely reflecting pandemic-related changes in elective procedures, demand for anesthesia, and pediatric respiratory admissions [17]. In addition, data for 2024 covered only January through September, and the lower absolute number of administration records observed in that year should therefore not be directly compared with complete calendar years.

The predominance of ICU admissions, especially in cardiac and surgical units, likely reflects the clinical complexity and illness severity of patients requiring opioid therapy. In this setting, naloxone-identified episodes frequently involved cardiorespiratory manifestations, including bradycardia, reduced SpO₂, and apnea. However, the distribution of these episodes across hospital units should not be interpreted as evidence of differences in event rates or risk between units. Hospital-unit information was available only for patients undergoing naloxone-triggered medical record review, whereas the pharmacy dataset did not provide the hospital unit in which opioid use occurred. Therefore, the number of opioid-treated patients within each hospital unit was unavailable, precluding calculation of unit-specific event rates. Higher susceptibility in those under 2 years may be related to ongoing maturation of neurophysiological and homeostatic regulatory mechanisms that can modulate responses to opioid exposure [18].

Consistent with the findings of the present pediatric center evaluation, Li et al. [19]. described adverse effects associated with postoperative analgesic use, including morphine and hydromorphone, in pediatric patients, with drowsiness and nausea reported as the most common events. Similarly, Tsampalieros et al. [20]. identified postoperative respiratory adverse events following morphine administration in children with obstructive sleep-disordered breathing and obstructive sleep apnea, with increased risk observed among patients with cardiac comorbidities, aligning with the patterns observed in the studied cohort. In another study, Cheng et al. [21]. evaluated intranasal fentanyl for procedural analgesia in preterm infants and reported respiratory adverse events in two of 30 patients, including respiratory deterioration with apnea and episodes of oxygen desaturation. Regarding methadone, a systematic review by Dervan et al. [22]. identified QTc prolongation and excessive sedation as notable adverse effects, as well as withdrawal symptoms. Outcomes varied across studies, with standardized tapering protocols associated with reduced withdrawal incidence, although discontinuation could still precipitate withdrawal in susceptible patients.

Although opioid use in children can reduce morbidity by enabling adequate analgesia and sedation, it may also be associated with adverse outcomes, including prolonged mechanical ventilation, increased use of total parenteral nutrition, and longer hospital stays. These factors may contribute to neurological alterations, such as impaired neurodevelopment, reduced cerebellar growth, developmental disabilities, and difficulties with social functioning [23,24]. Prolonged opioid exposure in children admitted to ICU has also been linked to potential long-term developmental consequences, including poorer cognitive and motor outcomes, particularly among preterm infants, and may complicate pain management strategies [25]. Although long-term neurodevelopmental and behavioral consequences of opioid exposure have been discussed in pediatric populations, the present study was not designed to evaluate long-term outcomes. The present findings are restricted to short-term clinical manifestations documented around naloxone administration [26].

Another factor that may contribute to increased susceptibility to opioid effects is the patient’s underlying comorbidity profile, which may act as an effect modifier, increasing vulnerability to opioid-related adverse events. In this retrospective analysis, congenital heart defects represented the most prevalent underlying conditions. Opioid use in cardiac patients warrants careful consideration, as certain opioids have been associated with QT-interval prolongation and potential proarrhythmic effects [27]. A study conducted by Weeke et al. [28], which evaluated patients with confirmed long QT syndrome receiving proarrhythmic pharmacotherapy, demonstrated that tramadol may constitute a potential risk factor for malignant ventricular arrhythmias, including torsades de pointes.

Naloxone was frequently associated with symptom resolution in this cohort; however, partial or absent responses observed in some cases may reflect the clinical complexity of these patients, multiple drug exposures, or other factors that could not be fully characterized in this retrospective analysis. Mortality related to underlying conditions, as well as the occurrence of withdrawal symptoms, although infrequent, highlights the importance of comprehensive patient monitoring, including careful evaluation of social and pharmacological histories, even in pediatric populations [25]. Given the use of naloxone as a screening trigger, the number of identified episodes should not be interpreted as the total burden of opioid-related adverse events. Some events may have been managed without naloxone or incompletely documented.

These findings highlight the importance of standardized documentation of naloxone indication, opioid exposure, clinical manifestations, timing, and response to reversal. Such documentation may improve opioid stewardship, facilitate pharmacovigilance, and help distinguish naloxone-identified suspected opioid-related adverse-event episodes from naloxone administrations not meeting criteria for adverse-event classification.

This study has some limitations. Opioid administration records were derived from pharmacy dispensing data and were used as a proxy for opioid utilization. Although records with documented return to pharmacy stock were excluded, the available data do not establish whether the dispensed medication was fully or partially administered to the patient at the bedside. The dataset contained incomplete or missing information, particularly regarding the indications for naloxone administration, which were not consistently documented in medical records. Consequently, cases in which naloxone was administered for indications other than opioid-related adverse events, such as during cardiopulmonary resuscitation or extubating, could not be reliably distinguished. Additionally, adverse events may have been underreported due to limited awareness or inadequate documentation practices. Episodes of opioid-related adverse events managed through alternative interventions without naloxone were also not captured by the screening strategy. The absence of data on naloxone use in surgical settings further restricted the scope of the analysis. Finally, this was a single-center study conducted at a tertiary, high-complexity pediatric hospital, and the characteristics of this patient population and institutional setting may limit the generalizability of the findings to other pediatric hospitals or healthcare settings.

In this tertiary pediatric hospital, naloxone-identified suspected opioid-related adverse-event episodes were uncommon relative to the total volume of opioid administration records and occurred predominantly in high-acuity settings, particularly cardiac and surgical ICUs. Fentanyl, morphine, and methadone were the most frequently documented opioids in these episodes, while respiratory, cardiovascular, and central nervous system manifestations were the most common clinical findings. Most episodes improved after naloxone administration, suggesting timely recognition and management in many cases. These findings support continued opioid stewardship, standardized documentation of naloxone indications, and risk-stratified monitoring protocols for complex pediatric inpatients (Figure 3).

**Figure 3:**
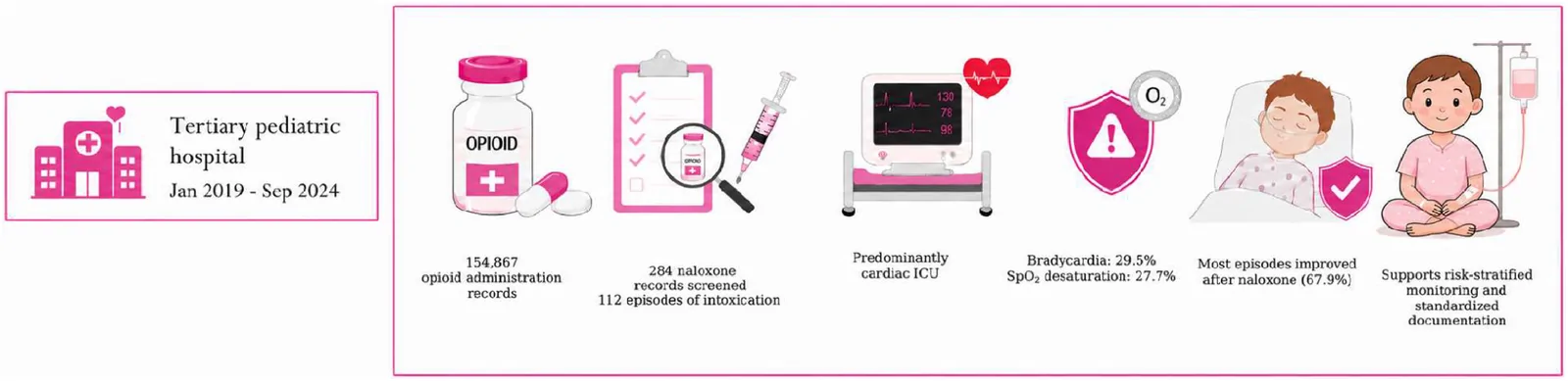
Clinical Characteristics and Outcomes of Naloxone-Associated Episodes.

## Funding sources

This study was financed in part by the Coordenação de Aperfeiçoamento de Pessoal de Nível Superior-Brasil (CAPES)-Finance Code 001, by the Instituto de Pesquisa Pelé Pequeno Príncipe (IPPPP), and by Fundação Araucária - Secretaria de Ciência, Tecnologia e Ensino Superior.

## Ethics approval

This study was performed in line with the principles of the Declaration of Helsinki. Approval was granted by the Ethics Committee of Hospital Pequeno Príncipe, Curitiba, Paraná, Brazil, under protocol number 6.599.719.

## Consent to participate

The Ethics Committee waived the requirement for informed consent due to the retrospective design and the use of de-identified hospital pharmacy and medical record data.

## Conflicts of interest

The authors declare no conflicts of interest.

## Data Availability

The datasets generated and/or analyzed during the current study are not publicly available due to ethical and privacy restrictions related to pediatric hospital records. Aggregated or de-identified data may be available from the corresponding author upon reasonable request and subject to institutional and ethics approval.

## Appendix Supplementary materials

Image, application 1

## References

[1] Lai B., Cunningham J., O’Keefe N., Chambers M.B., Clements C., Geyer H. (2025). Entering the fourth decade of the opioid crisis: an institution’s efforts to redefine opioid stewardship. Mayo Clin Proc.

[2] van Lemmen M.A., van Velzen M., Sarton E.Y., Dahan A., Niesters M., van der Schrier R.M. (2025). A comparison of intramuscular (Zimhi) and intranasal naloxone (Narcan) in reversal of fentanyl-induced apnea: a randomized, crossover, open-label trial. Nat Commun.

[3] Ki Y.J., Kim S.H., So K.Y. (2024). Effect of naloxone on anesthesia recovery among patients undergoing total intravenous anesthesia: a retrospective observational study. Med Biol Sci Eng.

[4] Saari T.I., Strang J., Dale O. (2024). Clinical pharmacokinetics and pharmacodynamics of naloxone. Clin Pharmacokinet.

[5] Gaw C.E., Gage C.B., Powell J.R., Ulintz A.J., Panchal A.R. (2025). Pediatric emergency medical services activations involving naloxone administration. Prehosp Emerg Care.

[6] Alban H., Ireifej N., D’Alessandro J., Jordan G., Lee R., Patricia N. (2025). Risk of hospital inpatient opioid overdose (RHINOO): a review of factors impacting naloxone administration in patients receiving opioids. Eur J Clin Pharmacol.

[7] Jacobson M., Powell D. (2025). Naloxone knowledge, carrying, purchase, and use. JAMA Netw Open.

[8] Phang K.G., Wahlquist A.E., Hayes G., Corrigan C., Basco W.T., Bundy D.G. (2024). Opioid dosing deviation and dose banding development in young hospitalized children. Hosp Pediatr.

[9] Amiot J., Bruge Ansel M.H., Bruyas M., Rinaudo M., Rulliat E., Gauthier J. (2024). Strategy to secure the neuraxial route in anaesthesia: risk mapping and feedback from the application of the ISO 80369-6 standard NRFit® connectors. Ann Pharm Fr.

[10] Aljalahma N., Alabbasi L., Alkoheji H., Almahmood H., Aldoseri A., Husain F. (2025). Accidental ingestion of intoxications (drugs and nondrug materials) among pediatric patients: a multicenter study in Bahrain royal medical services hospitals. J Toxicol.

[11] Smarczewski M.A., Osternack K.T., Garlet Q.I., Oliveira C.S., Mello R.G. (2021). Systematic review and single-centre toxicology study identified analgesics and benzodiazepines as the main causes of paediatric medication poisoning. Acta Paediatr.

[12] Carvalho R.M., de Magalhães-Barbosa M.C., Bianchi L.M., Rodrigues-Santos G., da Cunha A.J., Bastos F.I. (2023). Shift in hospital opioid use during the COVID-19 pandemic in Brazil: a time-series analysis of one million prescriptions. Sci Rep.

[13] Garcia J.B., Lopez M.P., Barros G.A., Muñiz H.G., Ahumada Olea M.A., Bonilla P. (2019). Latin American pain federation position paper on appropriate opioid use in pain management. Pain Rep.

[14] Maia L.O., Daldegan-Bueno D., Fischer B. (2021). Opioid use, regulation, and harms in Brazil: a comprehensive narrative overview of available data and indicators. Subst Abuse Treat Prev Policy.

[15] Vahos J., Rojas-Cortés R., Daza D., Osorio-Florez L.C., Macías Saint-Gerons D., Pastrana T. (2023). Barriers of access to opioid medicines within the context of palliative care in Latin America: the perception of health professionals. J Palliat Med.

[16] Carvalho R.M., Bastos F.I., de Magalhães-Barbosa M.C., Bianchi L.M., Robaina J.R., Rodrigues-Santos G. (2025). A time-series analysis of the use of opioid medications in a network of Brazilian hospitals, 2009-2020. Lancet Reg Health Am.

[17] Vásquez-Hoyos P., Diaz-Rubio F., Monteverde-Fernandez N., Jaramillo-Bustamante J.C., Carvajal C., Serra A. (2021). Reduced PICU respiratory admissions during COVID-19. Arch Dis Child.

[18] Steinbauer P., Kühnl J., Pichler K., Stummer S., Klebermass-Schrehof K., Deindl P. (2025). Cognitive, motor, and behavioral outcomes in preterm infants exposed to opioids. Pediatr Res.

[19] Li M.M., Larche C.L., Vickers K., Vigouroux M., Ingelmo P.M., Hovey R. (2022). Experience and management of the adverse effects of analgesics after surgery: a pediatric patient perspective. J Patient Exp.

[20] Tsampalieros A., Murto K., Barrowman N., Vaillancourt R., Bromwich M., Monsour A. (2022). Opioid dose and postoperative respiratory adverse events after adenotonsillectomy in medically complex children. J Clin Sleep Med.

[21] Cheng C., Tabbara N., Cheng C., Shah V. (2022). Intranasal fentanyl for procedural analgesia in preterm infants. Front Pain Res (Lausanne).

[22] Dervan L.A., Yaghmai B., Watson R.S., Wolf F.M. (2017). The use of methadone to facilitate opioid weaning in pediatric critical care patients: a systematic review of the literature and meta-analysis. Paediatr Anaesth.

[23] Keane O.A., Ourshalimian S., Lakshmanan A., Lee H.C., Hintz S.R., Nguyen N. (2024). Institutional and regional variation in opioid prescribing for hospitalized infants in the US. JAMA Netw Open.

[24] Selvanathan T., Miller S.P. (2024). Brain health in preterm infants: importance of early-life pain and analgesia exposure. Pediatr Res.

[25] Oschman A., Rao K. (2024). Challenges in management of refractory pain and sedation in infants. Front Pharmacol.

[26] Ajayi A.F., Oyowvi M.O., Akano O.P., Okeleji O.L., Adedeji B.O., Ajayi L.O. (2025). Unveiling the physiological systemic consequences of adolescent opioid use: a systematic review. J Child Adolesc Subst Use.

[27] Hu J., Song Y., Huang X., Li C., Jin X., Cen L. (2024). Opioids-induced long QT syndrome: a challenge to cardiac health. Cardiovasc Toxicol.

[28] Weeke P.E., Kellemann J.S., Jespersen C.B., Theilade J., Kanters J.K., Hansen M.S. (2019). Long-term proarrhythmic pharmacotherapy among patients with congenital long QT syndrome and risk of arrhythmia and mortality. Eur Heart J.

