# Supplementary material for: Opioid use and naloxone-identified suspected opioid-related adverse events in a tertiary pediatric hospital: a retrospective study
Source: J Pediatr (Rio J). 2026 Oct 30;103(1):101628. doi: 10.1016/j.jped.2026.101628 (PMC13743387; doi:10.1016/j.jped.2026.101628)
Supplement: Supplementary file 1 [file mmc1.docx]

**JPED-D-26-00300**

**Supplementary material**

**Opioid Use and Naloxone-Identified Suspected Opioid-Related Adverse Events in a Brazilian Tertiary Pediatric Hospital: A Retrospective Study**

**Figure S1** Annual opioid administration records from January 2019 to September 2024.

**Figure S2** Opioid administration records medications from January 2019 to September 2024, highlighting the most frequently dispensed drugs during the study period.

**Figure S3** Age distribution of patients receiving dispensed opioid medications between January 2019 and September 2024.

**Figure S4** Age distribution of patients who received naloxone. Colors represent event classification: pink, purple, blue, and green indicate suspected opioid-related adverse events, and cyan indicates presence of opioid-related adverse events.

**Table S1** Yearly opioid administration records volume across the study period (January 2019 to September 2024).

|  | **Year of dispensing** | | | | | | |
| --- | --- | --- | --- | --- | --- | --- | --- |
| **Drug** | **2019** | **2020** | **2021** | **2022** | **2023** | **2024** | **Total** |
| Afentanila 0.544mg/mL | 239 | 116 | 106 | 133 | 120 | 76 | 790 |
| Transdermal Buprenorphine 5mg | 0 | 0 | 2 | 0 | 0 | 1 | 3 |
| Fentanyl | 12,996 | 9309 | 10,468 | 11,446 | 12,887 | 9892 | 66,998 |
| Transdermal Fentanyl 2.1mg | 0 | 0 | 0 | 0 | 1 | 5 | 6 |
| Transdermal Fentanyl 4.2mg | 0 | 0 | 0 | 0 | 0 | 3 | 3 |
| Transdermal Fentanyl 8.4mg | 0 | 1 | 0 | 0 | 0 | 0 | 1 |
| Methadone 10mg | 5296 | 2774 | 2521 | 2430 | 3496 | 3101 | 19,618 |
| Methadone 10mg/mL | 539 | 672 | 650 | 689 | 760 | 659 | 3969 |
| Methadone 5mg | 0 | 0 | 0 | 0 | 205 | 0 | 205 |
| Morphine 0,2mg/mL | 113 | 79 | 103 | 96 | 88 | 62 | 541 |
| Morphine 10mg | 38 | 67 | 229 | 196 | 346 | 136 | 1012 |
| Morphine | 11,075 | 8373 | 9267 | 10,736 | 11,163 | 8669 | 59,283 |
| Naloxone 0.4mg/mL | 63 | 34 | 47 | 58 | 35 | 47 | 284 |
| Remifentanyl 2mg | 362 | 357 | 319 | 382 | 420 | 314 | 2154 |
| Total | 30,721 | 21,782 | 23,712 | 26,166 | 29,521 | 22,965 | 154,867 |

**Table S2** Individual-level data for naloxone-identified suspected opioid-related adverse-event episodes included in this study.

| **Year** | **Patient** | **Opioid** | **Underlying condition/reason** | **ICD-10** | **Unit of hospitalization** | **Documented clinical manifestation** | **Short-term outcome after naloxone administration** |
| --- | --- | --- | --- | --- | --- | --- | --- |
| 2019 | A1 | Fentanyl | Laryngomalacia | Q31.5 | ICU | Rigidity thoracic and bradycardia | Symptoms improved |
| 2019 | A2 | Fentanyl | Down syndrome, West syndrome, respiratory failure - bronchospasm and stridor | Q90.9, G40.4, J96.9, J98.01, R06.1 | General ICU | Hypotension and bradycardia | Symptoms improved |
| 2019 | A3 | Morphine | Enlarged right ventricle | I51.7 | Surgical ICU | Sedation without response to painful stimuli | Symptoms improved |
| 2019 | A4 | Fentanyl | Osteogenesis imperfecta | Q78.0 | Neonatal ICU | Bradycardia and oxygen desaturation | Symptoms improved |
| 2019 | A5 | Fentanyl | Tricuspid atresia type IB, POT shunt de Blalock-Taussig | Q22.4 | Cardiac ICU | Apnea | Symptoms improved |
| 2019 | A5 | Fentanyl | Tricuspid atresia type IB, POT shunt de Blalock–Taussig | Q22.4 | Cardiac ICU | Rigidity thoracic, respiratory distress, and oxygen desaturation | Sustained respiratory effort |
| 2019 | A6 | Fentanyl | VSD | Q21.0 | Cardiac ICU | Apnea | Symptoms improved |
| 2019 | A7 | Fentanyl | POT Tetralogy of Fallot | Q21.3 | Surgical ICU | Sedation, stridor, and oxygen desaturation | Symptoms improved |
| 2019 | A8 | Fentanyl | Newborn small for gestational age, neonatal toxoplasmosis, bilateral grade I peri-/ IVH, microcephaly, sepsis secondary to meningitis, hypertrophic pyloric stenosis, intrauterine drug exposure | P05.0, P37.1, P52.0, Q02, P04.4 | Surgical ICU | Increased heart rate with diminished heart sounds | Symptoms improved |
| 2019 | A9 | Fentanyl | HLHS | Q23.4 | Cardiac ICU | Apnea | Symptoms improved |
| 2019 | A10 | Fentanyl, Tramadol | Acute myeloid leukemia, myelomonocytic | C92.4 | General ICU | Gasping respirations accompanied by deterioration of respiratory pattern and oxygen desaturation. | Death due to the underlying disease |
| 2019 | A11 | Fentanyl, Morphine | Complete AVSD, Rastelli type A associated with PDA | Q21.2, Q25.0 | Cardiac ICU | Oxygen desaturation, decreased level of consciousness, and bradycardia. | Symptoms improved |
| 2019 | A12 | Methadone, Morphine | Moderate to large trabecular VSD, large large ostium secundum atrial septal defect, mild mitral stenosis, bicuspid aortic valve, significant pulmonary hypertension | Q21.0, Q21.1 | Cardiac ICU | Apnea with bradycardia | Symptoms improved |
| 2019 | A13 | Methadone | Dystonic spastic quadriplegic cerebral palsy, microcephaly, West syndrome, GERD, esophageal stricture, gastrostomy tube dependence | G80.3, Q02, G40.4, K21.0 | Surgical ICU | Respiratory depression | Symptoms improved |
| 2019 | A14 | Fentanyl | History of intrauterine growth restriction, hydrocephalus, necrotizing enterocolitis | P05.1, Q03.9, P77 | ICU | Oxygen desaturation, reduced lung expansion, and CPA | Partial improvement |
| 2019 | A15 | Morphine | Seckel syndrome, malnutrition, scoliosis, pulmonary compromise | Q87.1 | General ICU | Oxygen desaturation, respiratory deterioration, and drowsiness. | Symptoms improved |
| 2019 | A16 | Fentanyl | Tricuspid atresia type IB | Q22.4 | Cardiac ICU | Bradycardia and bradypnea | Symptoms improved |
| 2019 | A17 | Methadone, Morphine | Pneumonia, respiratory failure, bronchiolitis | J18.9, J96.9, J21.0 | Surgical ICU | Bradycardia, hypoxia, cyanosis, severe respiratory distress, and chest wall rigidity | Partial improvement |
| 2019 | A18 | Fentanyl | Late preterm newborn, corrected laryngomalacia, POT epiglottectomy, cow’s milk protein allergy, moderate cerebral distress | P07.3, Q31.5, K52.2, P21.9 | Neonatal ICU | Reduced chest expansion | Symptoms improved |
| 2019 | A18 | Fentanyl | Late preterm newborn, corrected laryngomalacia, POT epiglottectomy, cow’s milk protein allergy, moderate cerebral distress | P07.3, Q31.5, K52.2, P21.9 | Neonatal ICU | Seizure and worsening of respiratory effort | Symptoms improved |
| 2019 | A19 | Fentanyl | Preterm newborn, duodenal atresia, laparotomy, intestinal necrosis and perforations, jejunostomy, short bowel, ileocecal valve resection | P07.3, Q41.0, K63.1, K91.2 | Surgical ICU | Oxygen desaturation, perioral cyanosis, globally reduced breath sounds, infrequent scattered wheezing, and chest wall rigidity | Symptoms improved |
| 2019 | A20 | Fentanyl, Morphine | Glucose-6-phosphate dehydrogenase deficiency, mitochondrial disease complex 3, renal tubulopathy (tubular acidosis) | D55.0, E88.40, N25.8 | General ICU | Shallow respirations and bradypnea | Symptoms improved |
| 2019 | A21 | Fentanyl, Morphine | Late preterm newborn, facial necrosis, cerebral infarction, cardiac arrest, fungal sepsis, vembrosis, left pleural effusion, gastrostomy | P07.3, P05.0, L98.4, I63.9, I46.9, | Neonatal ICU | Oxygen desaturation followed by bradycardia and cardiac arrest | Tracheostomy due to lack of improvement |
| 2019 | A22 | Methadone, Morphine | Obstructed (restrictive) infracardiac TAPVR associated with PDA, ASD, and significant pulmonary hypertension | Q26.2, Q25.0, Q21.1, I27.2 | Surgical ICU | Chest injuries associated with decreased thoracic expansion and bradycardia | No-symptoms improved |
| 2020 | A23 | Fentanyl | Tetralogy of Fallot | Q21.3 | Cardiac ICU | Apnea | Symptoms improved |
| 2020 | A24 | Fentanyl | Post-myocarditis dilated cardiomyopathy | I42.0, I40.9 | Cardiac ICU | Apnea | Symptoms improvement and agitation |
| 2020 | A25 | Morphine | Epilepsy, cerebellar tumor | G40.9, | Surgical ICU | Stridor | Partial improvement |
| 2020 | A26 | Fentanyl, Morphine | Aortic valve stenosis | I35.0 | Cardiac ICU | Bradypnea | Symptoms improved |
| 2020 | A27 | Fentanyl, Morphine | Perimembranous VSD with hemodynamic repercussions | Q21.0, | Cardiac ICU | Three brief apneic episodes with decreased responsiveness | Symptoms improved |
| 2020 | A28 | Morphine | Cerebral atrophy, malformation in upper limbs, laryngotracheomalacia and moderate pharyngeal dysphagia, ASD, epilepsy. | G31.8, Q74.0, Q31.8, R13.12, Q21.1, Q40.9 | Ward | Oxygen desaturation and stridor secondary to airway malacia | Symptoms improved |
| 2020 | A29 | Fentanyl | Duodenal atresia, intestinal malrotation | Q41.0, Q43.3 | Surgical ICU | Cardiopulmonary arrest with associated cyanosis, oxygen desaturation, and bradycardia | Partial improvement |
| 2020 | A30 | Fentanyl | Immature teratoma | B10.9 | Surgical ICU | Generalized tonic movements, decreased level of consciousness, and miosis | Partial improvement |
| 2020 | A31 | Fentanyl, Morphine | Small VSD, large ASD, tricuspid valve with septal leaflet displaced apically by the VSD closure mechanism | Q21.0, Q21.1, Q22.5 | Cardiac ICU | Chest rigidity and sedation | Symptoms improved |
| 2020 | A32 | Fentanyl | Critical valvular pulmonary stenosis evolving with significant double tricuspid lesion with predominant insufficiency | Q22.1, I36.1 | Cardiac ICU | Agitation | Symptoms improved |
| 2020 | A33 | Fentanyl | Left ventricular hypoplasia | Q23.4, | Cardiac ICU | Sedative withdrawal syndrome with bradycardia | Not informed |
| 2020 | A34 | Methadone | Mucopolysaccharidosis type II | E76.1 | Surgical ICU | Hypotension, bradycardia, and miotic pupils | Symptoms improved |
| 2020 | A35 | Morphine | Aortopulmonary window, two minimal trabecular VSD, FOP, mild pulmonary hypertension | Q25.4, Q21.0, I27.2, M61.1 | Cardiac ICU | Chest wall rigidity | Symptoms improved |
| 2020 | A36 | Fentanyl, Morphine | Double-inlet left ventricle with a hypoplastic right ventricle, ventriculoarterial discordance, a non-restrictive ventricular septal defect, mitral stenosis, status post pulmonary artery banding | Q20.3, Q22.6, Q21.0, Q23.2, | Cardiac ICU | Agitation, sinus tachycardia, and marked cyanosis progressing to apnea | Symptoms improved |
| 2020 | A37 | Methadone | Large ostium secundum atrial septal defect, moderate VSD, large PDA, pulmonary hypertension | Q21.1, Q21.0, Q25.0, I27.2 | Cardiac ICU | Reduced chest expansion with low tidal volume | Symptoms improved |
| 2020 | A38 | Fentanyl, Morphine | Cerebral palsy due to neonatal infection, bronchodysplasia, obesity, cow’s milk protein allergy | G80.9, P27.1, K52.2 | General ICU | Decreased level of consciousness, unresponsiveness, absent palpable pulses, prolonged capillary refill, cold extremities, and tachypnea | No-symptoms improved |
| 2020 | A39 | Methadone | Ebstein's Anomaly, supraventricular tachycardia | Q22.5, I47.1 | Cardiac ICU | Desaturation and bradycardia | Symptoms improved |
| 2020 | A40 | Methadone | Dilated cardiomyopathy | I42.0, | Cardiac ICU | Profuse vomiting, marked drowsiness (minimally responsive to stimuli), and pallor | Symptoms improved |
| 2020 | A41 | Codeina | Matched related donor hematopoietic stem cell transplantation for acute lymphoblastic leukemia in first complete remission | C91.0 | Surgical ICU | Drowsiness | Symptoms improved |
| 2021 | A42 | Fentanyl | Posterior mediastinal tumor | D15.2 | Cardiology ward | Pallor, cardiac arrest, ventricular fibrillation | Death due to the underlying disease |
| 2021 | A43 | Morphine | Brain tumor | C71.X | Surgical ICU | Decreased level of consciousness, drowsiness, bradypnea, unresponsiveness to verbal commands, and absence of eye opening | Symptoms improved |
| 2021 | A44 | Fentanyl | Dilated cardiomyopathy resulting from anomalous coronary artery | I42.0 | Pediatric ward | Decreased level of consciousness, bradycardia, and responsiveness only to painful stimuli | Symptoms improved |
| 2021 | A45 | Fentanyl | POT aortic valvuloplasty with bicuspid aortic valve with mild residual double lesion, parachute mitral valve with moderate stenosis, small subaortic VSD with right coronary cusp prolapse, moderate pulmonary hypertension | Q23.1, I35.2, Q23.4, Q23.2, Q21.0, I34.1, I27.2 | Cardiac ICU | Bradypnea | Symptoms improved |
| 2021 | A46 | Fentanyl | Tricuspid atresia type II-B with transposition of the great arteries, a large VSD, non-restrictive ASD, and severe pulmonary outflow tract obstruction | Q20.3, Q21.0, Q21.1, Q22.1 | Cardiac ICU | Altered consciousness | Symptoms improved |
| 2021 | A47 | Fentanyl, Morphine | Truncus type IV with pulmonary atresia and pulmonary flow through systemic-pulmonary collateral | Q20.0, Q22.0, Q25.6 | Cardiac ICU | Apnea | Symptoms improved |
| 2021 | A48 | Morphine | Double-outlet right ventricle large, widely committed VSD, persistent left superior vena cava, significant pulmonary hypertension, severe subaortic stenosis with a fibromuscular component, and mild aortic regurgitation | Q20.4, Q21.0, Q26.4, I27.2, Q24.3, I35.1 | Cardiac ICU | Skin pallor and bradypnea | Symptoms improved |
| 2021 | A49 | Fentanyl | VSD with large outflow tract ASD, moderate pulmonary stenosis, oriental four-fibroid syndrome. | Q21.0, Q22.1, Q21.3 | Cardiac ICU | Apnea and bradycardia | Symptoms improved |
| 2021 | A50 | Fentanyl, Methadone | VSD, ASD | Q21.0, Q21.1 | Cardiac ICU | Sedation and bradycardia | Symptoms improved |
| 2021 | A51 | Morphine | Caustic soda poisoning | T54.3 | Surgical ICU | Respiratory distress, oxygen desaturation, and drowsiness | Symptoms improved |
| 2021 | A52 | Fentanyl | Epileptic encephalopathy (protocadherin-19 gene mutation), Cow’s milk protein allergy | Q40.4, Q40.8, Q52.2 | ICU | Cardiopulmonary arrest, absence of spontaneous movement, and reduced motor activity | Symptoms improved |
| 2021 | A53 | Fentanyl, Morphine | Large ostium secundum ASD, large perimembranous VSD | Q21.1, Q21.0 | Cardiac ICU | Drowsiness | Symptoms improved |
| 2021 | A54 | Fentanyl | Tricuspid atresia with double-outlet right ventricle, hypoplastic left ventricle, an anteriorly positioned aorta, severe pulmonary outflow tract obstruction | Q22.4, Q20.4, Q23.4, Q20.3, Q22.1 | Cardiac ICU | Respiratory distress, tachypnea, decreased responsiveness, and marked fatigue | Symptoms improved |
| 2021 | A55 | Methadone | Osteogenesis imperfecta, gastrostomy with recurrent ostium infections, multiple bone fractures | Q78.0, T02.9 | Emergency care | Bradypnea, shallow breathing, and unresponsiveness, episode of ocular eversion, miosis, bradypnea, and desaturation, seizure | Symptoms improved |
| 2021 | A56 | Fentanyl | Hydranencephaly, status post ventriculoperitoneal shunt and external ventricular drain | Q04.3, Z96.2, | Neurosurgery | Chest wall rigidity, oxygen desaturation, bradycardia, and seizure | Symptoms improved |
| 2021 | A57 | Fentanyl | POT AVSD, mixed mitral valve disease (stenosis and regurgitation) | Q21.2, I34.2, | Cardiac ICU | Apnea and oxygen desaturation | Symptoms improved |
| 2021 | A58 | Fentanyl | PDA, grade III intraventricular hemorrhage, bronchopulmonary dysplasia | Q25.0, B97.4, P97.1 | ICU | Chest wall rigidity | Reconnected to mechanical ventilation |
| 2021 | A59 | Fentanyl | Preterm newborn, respiratory distress syndrome, suspected/possible neonatal infection, perinatal asphyxia, pulmonary hypertension, acute respiratory failure PDA, central apnea, myocarditis, bicuspid aortic valve, pulmonary hypertension | P07.2, P05.0, P22.0, N17.9, Q25.0, R06.81, I40.9, I27.2 | Infectious diseases ward | Chest wall rigidity | Symptoms improved |
| 2022 | A60 | Fentanyl, Morphine | Critical pulmonary valve stenosis | Q22.1 | Cardiac ICU | Apnea post-extubation | Symptoms improved |
| 2022 | A61 | Fentanyl | Kenny–Caffey syndrome, gracile bone syndrome (skeletal dysplasia), hypoparathyroidism, hyperphosphatemia, symptomatic hypocalcemia, hereditary spherocytosis, normocytic anemia, ocular motor cranial nerve immaturity, left retinal hemorrhage, seizures/epilepsy, multiple fractures, ventricular dilation | Q77.9, E20.0, E83.51, T02.9, D58.0, K92.2, H49.3, H35.6, R56.8, T02.0, Q03.8 | Specialized surgical ICU | Oxygen desaturation and chest wall rigidity | Improvement in the symptoms, followed by death due to the underlying condition. |
| 2022 | A62 | Fentanyl | Acute viral bronchiolitis | Q90.9 | Emergency care | Chest wall rigidity | Symptoms improved |
| 2022 | A63 | Methadone, Morphine | Bannayan-Riley-Ruvalcaba syndrome (PTEN gene mutation), asthma | Q87.8, J45.9 | Neurology ward | Apnea associated with oxygen desaturation, nausea, and vomiting | Symptoms improved |
| 2022 | A64 | Morphine, Morphine | Encephalopathy | G93.4 | Pediatric neurology | Bradypnea, decreased level of consciousness, pyrexia, and vomiting | Slight clinical improvement, subsequently transferred to the general ICU following an episode of decreased level of consciousness |
| 2022 | A65 | Fentanyl, Methadone | Preterm newborn, pulmonary malformation | P07.3, P05.1, Q33.8 | Neonatal ICU | Agitation, signs of opioid dependence, chest wall rigidity | Withdrawal syndrome |
| 2022 | A66 | Fentanyl | Preterm newborn, anhydramnios, bilateral hydronephrosis, multicystic dysplastic right kidney | P07.3, Q61.4, Q62.1, N17.9 | Specialized surgical ICU | Apnea and central cyanosis | Symptoms improved |
| 2022 | A67 | Fentanyl, Morphine | Complete AVSD, Rastelli type C, Down syndrome | Q21.2, Q90.0 | Cardiac ICU | Oxygen desaturation, worsening respiratory status, and hypoactivity | Symptoms improved |
| 2022 | A68 | Fentanyl, Morphine | Pulmonary stenosis, structural epilepsy, moderate intellectual disability | Q21.3, Q22.1 | Cardiac ICU | Bradycardia, sedation, and abnormal respiratory pattern | Symptoms improved |
| 2022 | A69 | Nafazolina | Exogenous intoxication | T50.9 | Emergency care | Naphazoline poisoning | Not informed, off-label use |
| 2022 | A70 | Morphine | Pulmonary hypertension crisis | E20.0 | Emergency care | Nausea, oxygen desaturation, diaphoresis, psychomotor agitation, and tachycardia | Death due to the underlying disease |
| 2022 | A71 | Fentanyl | Preterm infant, small for gestational age, right diaphragmatic hernia | P07.3, P05.0, Q79.0 | Neonatal ICU | Absent chest expansion | No-symptoms improved |
| 2022 | A72 | Fentanyl, Methadone | Severe asthma, respiratory failure, pneumonia | J45.5, J96.0, J18.9 | General ICU | Drowsiness, oxygen desaturation, poor responsiveness to stimulation, and decreased level of consciousness | Symptoms improved |
| 2022 | A73 | Fentanyl | Significant aortic coarctation | Q25.1 | Cardiac ICU | Apnea and bradycardia | Not specified, had previously experienced bradycardia after using fentanyl, with improvement after discontinuation of use |
| 2022 | A74 | Fentanyl, Morphine | Trignocephaly, postoperative craniosynostosis | Q75.0 | Surgical ICU | Apnea, cyanosis, oxygen desaturation, and bradycardia with preserved peripheral pulses | Symptoms improved |
| 2022 | A75 | Codeina (4cp) 30mg, Tramadol (9cp) 50mg | Suicide attempt | X84 | Emergency care | Paresthesia in the hands, blurred vision, and headache | Symptoms improved |
| 2022 | A76 | Morphine | Congenital toxoplasmosis, hydroanencephaly, COVID | P37.1, Q04.3, J96.0 | General ICU | Respiratory acidosis and hypopnea | Symptoms improved |
| 2022 | A77 | Opioid suspect intoxication | Exogenous intoxication | T50.9 | Emergency care | Bradycardia, decreased level of consciousness | Slight improvement in the condition, ICU admission. |
| 2022 | A78 | Fentanyl, Methadone , Morphine | Double inlet and outlet of a single ventricle, significant valvular pulmonary stenosis and moderate PDA | Q20.4, Q22.1, Q25.0 | Cardiac ICU | Apnea with decreased saturation and bradycardia | Partial improvement |
| 2022 | A79 | Fentanyl, Methadone, Morphine | Double outlet right ventricle, ASD, partial anomalous drainage of right ventricular posterior valves | Q20.1, Q21.1, Q26.2 | Cardiac ICU | Agitation, desaturation, eye deviation, tachycardia, respiratory effort with stridor | Partial improvement |
| 2022 | A80 | Methadone | Biliary atresia, liver transplant | Q44.2 | Surgical ICU | Bradycardia, cyanosis, and unresponsiveness | Symptoms improved |
| 2022 | A81 | Fentanyl, Methadone | Pulmonary atresia (imperforate pulmonary venous catheter) with intact interventricular septum, hypertrophic right ventricle, large PDA and small ASD with bidirectional flow | Q22.0, Q25.0, Q21.1, | Cardiac ICU | Bradycardia | Symptoms improved |
| 2022 | A82 | Methadone | Double-outlet right ventricle, ASD, large VSD, Glenn postoperative period | Q20.1, Q21.1, Q21.0 | Cardiac ICU | Intense agitation alternating with drowsiness | Partial improvement |
| 2023 | A83 | Morphine | Teratoid/rhabdoid tumor of the cervical spinal cord with tetraparesis | C72.0, Q83.4 | ICU | Respiratory effort, cold extremities, and drowsiness | Death due to the underlying disease |
| 2023 | A84 | Methadone | Car accident | V89.2 | Surgical ICU | Decreased level of consciousness | No-symptoms improved |
| 2023 | A85 | Fentanyl, Morphine | Sickle cell anemia with previous strokes | D57.0, I69.3 | Surgical ICU | Decreased level of consciousness, isophotoreactive pupils, worsening respiratory pattern with significant external retention, suprasternal and intercostal retractions | Death due to the underlying disease |
| 2023 | A86 | Morphine | Appendectomy | Z90.49 | General ICU | Seizure, respiratory distress, tachycardia, cyanosis of extremities, and tremor | Symptoms improved |
| 2023 | A87 | Fentanyl, Methadone, Morphine | Severe coarctation of the aorta | Q25.1 | Cardiac ICU | Chest wall rigidity and transient bradycardia | Symptoms improved |
| 2023 | A88 | Fentanyl | Epilepsy | G40.9 | Cardiac ICU | Nausea, vomiting, apnea, and hyporesponsiveness | Symptoms improved |
| 2023 | A89 | Fentanyl | Preterm infant, respiratory distress syndrome, exposure to syphilis, jaundice, inguinal hernia, intestinal perforation, postoperative typhlectomy and terminal ileostomy, thrombocytopenia, severe pulmonary hypertension | P07.3, P05.1, P22.0, P35.0, P59.9, | Neonatal ICU | Chest wall rigidity, bradycardia, and respiratory acidosis | Death due to the underlying disease |
| 2023 | A90 | Fentanyl | Tricuspid atresia | Q22.4 | Cardiac ICU | Chest wall rigidity with a drop in saturation to 60% | Symptoms improved |
| 2023 | A91 | Morphine | Preterm infant, Charge syndrome, bilateral choanal atresia, cerebral atrophy, deafness, blindness (bilateral iris coloboma), bronchopulmonary dysplasia | P07.3, Q87.0, Q30.0, G31.1, H90.3, Q13.1, P27.1 | Specialized surgical ICU | Drowsiness | Symptoms improved |
| 2023 | A92 | Fentanyl, Morphine | Left ventricular hypoplasia, subaortic VSD, aortic atresia, aortic hypoplasia, pulmonary hyperflow, right ventricular dysfunction hybrid POT | Q20.4, Q21.0, Q23.0, Q25.4, I27.81, I51.8, | Emergency care | Sudden respiratory effort, grunting, and sporadic cough | Death due to the underlying disease |
| 2023 | A93 | Fentanyl | Hydrocephalus, valve dysfunction, seizure | G91.9, T85.0, R56.9 | Surgical ICU | Drowsiness and vomiting, deep sedation | Death due to the underlying disease |
| 2023 | A94 | Fentanyl, Morphine | Cerebral palsy, epilepsy, pneumonia, electrolyte imbalance, thrombocytopenia | G80.9, G40.9, J18.9, E87.9 | Surgical ICU | Drowsiness, miotic pupils, and bradycardia | Symptoms improved |
| 2023 | A95 | Morphine | Type 3 laryngotracheoesophageal cleft, extrinsic tracheal compression, bronchiolitis, pulmonary artery obstruction | P05.0, Q39.0, J39.8 | Pediatric neurology | Drop in oxygen saturation, bilaterally reduced lung sounds, desaturation, bradycardia, and seizure | Partial improvement |
| 2024 | A96 | Fentanyl | Congenital heart disease, VSD | Q21.0 | Cardiac ICU | Oxygen desaturation, seizure, absence of chest expansion, and bradycardia | Symptoms improved |
| 2024 | A97 | Fentanyl | Heart disease | Q24.9 | Cardiac ICU | Thoracic rigidity, oxygen desaturation, and bradycardia | Death due to the underlying disease |
| 2024 | A98 | Naridrin | Naphazoline poisoning | T50.9 | Emergency care | Off-label use | Not informed, off-label use |
| 2024 | A99 | Codeina | Hodgkin's lymphoma | C81.9 | Emergency care | Decreased level of consciousness, eye opening to painful stimulus, isochoric mydriatic and bradyreactive pupils | Symptoms improved |
| 2024 | A100 | Morphine | POT cranioplasty | Z98.890 | Pediatric ICU | Drowsiness, miotic pupils, oxygen desaturation with tachycardia and seizure | Symptoms improved |
| 2024 | A101 | Morphine | Type I diabetes, diabetic ketoacidosis | E10.1 | General ICU | Tachypnea with moderate intercostal and subcostal retractions | No-symptoms improved |
| 2024 | A102 | Fentanyl | Trunk type I, significant pulmonary hypertension | Q20.0, I27.0 | Cardiac ICU | Bradycardia | Death due to the underlying disease |
| 2024 | A103 | Methadone 10mg/mL | Tetralogy of Fallot with significant pulmonary embolism and ASD | Q21.3, Q21.1, Q22.1 | Cardiac ICU | Apnea | Symptoms improved |
| 2024 | A104 | Depakene | Depakene poisoning | T50.9 | Emergency care | Not informed | Not informed, off-label use |
| 2024 | A105 | Methadone | Post-operative choledochal cyst resection | Q44.4 | Surgical ICU | Miotic pupils and oxygen desaturation | Symptoms improved |
| 2024 | A106 | Fentanyl, Methadone | Dilated cardiomyopathy | I42.0 | Cardiac ICU | Bradycardia | Symptoms improved |
| 2024 | A107 | Fentanyl, Morphine | ASD | Q21.1 | Cardiac ICU | Drowsiness with respiratory effort, hypoventilation, and hypotension | Symptoms improved |
| 2024 | A108 | Fentanyl | PDA, asthma | Q25.0, J45.9 | Cardiac ICU | Apnea | Symptoms improved |
| 2024 | A109 | Fentanyl | Pulmonary atriovenous fistula | Q26.8 | Cardiac ICU | Bradypnea, drowsiness, irritability, cyanosis, and mild dyspnea | Symptoms improved |
| 2024 | A110 | Fentanyl, Methadone, Morphine | Pulmonary hypertension of left atrial enlargement, right chamber enlargement | I27.2, I51.7 | Cardiac ICU | Bradycardia, apnea and desaturation | Symptoms improved |

ASD, atrial septal defect; AVSD, atrioventricular septal defect; CPA, cardiopulmonary arrest; FOP, fibrodysplasia ossificans progressiva; GERD, gastroesophageal reflux disease; ICU, intensive care unit; PDA, patent ductus arteriosus; POT, post-operative; TAPVR, total anomalous pulmonary venous return; VSD, ventricular septal defect.
